# Gastroesophageal Reflux Characteristics and Patterns in Patients with Idiopathic Subglottic Stenosis

**DOI:** 10.1155/2018/8563697

**Published:** 2018-06-11

**Authors:** Hongfei Fang, Don C. Codipilly, Karthik Ravi, Dale C. Ekbom, Jan L. Kasperbauer, Magnus Halland

**Affiliations:** ^1^Department of Medicine, Mayo Clinic, Rochester, MN, USA; ^2^Division of Gastroenterology and Hepatology, Mayo Clinic, Rochester, MN, USA; ^3^Department of Otorhinolaryngology, Mayo Clinic, Rochester, MN, USA

## Abstract

**Introduction:**

Idiopathic subglottic stenosis represents a spectrum of subglottic disease without a clear underlying cause. Prior studies have implicated a pathogenic role of gastroesophageal reflux disease in idiopathic subglottic stenosis. The aim of this study was to examine the presence and pattern of gastroesophageal reflux in a large cohort of patients with idiopathic subglottic stenosis at a tertiary referral center.

**Methods:**

We performed a retrospective review of patients with idiopathic subglottic stenosis from January 2010 to December 2016 who had undergone combined pH impedance testing. Patients with prior gastric or esophageal surgery were excluded. Data obtained included esophageal acid exposure times, number of reflux events, patient position during reflux events (defined as upright, supine, or mixed), body mass index, and the presence of proton pump inhibitor therapy.

**Results:**

159 patients with the idiopathic subglottic stenosis were identified, of whom 41 had undergone esophageal pH impedance testing. 40 (97.6%) were women, with a mean age of 54.8 (range 31–79) years and BMI of 31.0 (range 17–55). Overall, 19 (46.3%) patients were found to reflux as confirmed by abnormal esophageal acid exposure or abnormal number of reflux events. 15 of the 19 patients with reflux had predominantly upright gastroesophageal reflux disease, whereas 2 had supine and 2 mixed reflux.

**Discussion:**

In patients with idiopathic subglottic stenosis who underwent evaluation by combined pH impedance, close to half were found to have gastroesophageal reflux disease. The majority of gastroesophageal reflux occurred while the patients were in the upright position.

## 1. Introduction

Subglottic stenosis is a disease that involves narrowing of the airway around the cricoid cartilage which can lead to airway compromise. There are various causes of subglottic stenosis, including trauma, neoplastic, infectious, systemic inflammatory disorders or congenital anomalies [1]. However, despite extensive evaluation, many cases of subglottic stenosis are deemed idiopathic. Idiopathic subglottic stenosis (ISS) represents a spectrum of disease with an unclear underlying pathophysiology and a significant challenge for clinical management.

Proposed factors from retrospective and observational studies include a role of hormones [2], growth factors [3], aberrant immune activation [4], and even bacteria [4] contributing to the disease. However, several studies have implicated a potential role of gastroesophageal reflux disease [5–7] (GERD). The leading hypothesis is that reflux of gastric contents into the upper airway contributes to the development of ISS. For example, in a prior study of 33 patients with laryngeal or tracheal stenosis, abnormal esophageal acid exposure was found in 56% [8]. Of note, among the 26 patients in this study who were tested off acid suppression, 23 (88%) had abnormal esophageal acid exposure. In another study which included 74 pediatric patients with subglottic stenosis using both upper and lower esophageal pH probe testing [9], it was noted that 32% of patients had pH of <4.0 in the lower esophagus for greater than 10% of study time, while only 20% of patients tested had pH of <4.0 in the upper esophagus greater than 3% of study time [9]. However, this study included 11 patients who had undergone prior Nissen fundoplication and 23 with bronchopulmonary dysplasia. In another prospective study of 22 patients with ISS, 7 of 10 patients were found to have reflux by 24-hour pH testing [5]. In addition, 59% of patients had detectable pepsin in laryngeal/tracheal tissue whereas none was found in the control group. However, there was no correlation between presence of reflux and pepsin. In a retrospective review of 37 ISS patients undergoing interval endoscopic balloon dilation, a higher prevalence of gastroesophageal reflux symptoms was noted in the medical records of these patients compared to the general population, but no objective tests for GERD were performed [10]. Other limited studies include 7 patients that had improvement of ISS after medical treatment for gastroesophageal reflux [6] and 5 of 7 patients with ISS exhibiting acid reflux through pH testing [7]. Finally, a study examining 109 patients evaluated in a laryngology clinic with suspected laryngopharyngeal reflux using dual pH with multichannel intraluminal impedance probes found proximal reflux exposure in 6 of 12 patients with subglottic stenosis [11]. However, in this study, it was not clear whether the subglottic stenosis was secondary in nature versus idiopathic.

Taken together, the prior studies suggest that GERD might be a factor in ISS. However, the current knowledge is limited by both significant heterogeneity in the diagnostic method used to diagnose GERD and limited sample size of the prior studies. In clinical practice, most patients with ISS receive an aggressive antireflux medication as part of treatment for subglottic stenosis [12]. Whether this impacts the disease course in ISS is unclear. Although these past studies have implicated a potential role of GERD in the development of ISS, the characterization of the pattern of reflux, that is, whether these patients have mixed, supine, or predominant upright reflux, is unclear. Whether adult patients with ISS have pathological GERD as defined by combined pH impedance testing is also unknown. Furthermore, with emerging concerns about long-term safety of proton pump inhibitor (PPI) therapy [13–15], the current clinical practice of empiric high-dose PPI therapy in this group can be questioned. As such, we aimed to examine the presence and pattern of gastroesophageal reflux disease in a large cohort of patients with ISS at a tertiary referral center.

## 2. Materials and Methods

### 2.1. Patient Population

We reviewed the electronic Mayo Clinic medical record and our own prospectively maintained internal subglottic stenosis database to identify cases of idiopathic subglottic stenosis. In this study, we only included patients with ISS who had undergone a pH impedance study. We excluded patients who had undergone prior gastric or esophageal surgery.

### 2.2. Assessment of Airway Symptoms

Symptoms of airway obstruction were obtained from chart review during their clinical visit closest to time of the esophageal pH study. These symptoms included shortness of breath and dyspnea. Symptoms were recorded as either present or absent.

### 2.3. Assessment of Reflux

Assessment for reflux occurred using a multichannel intraluminal impedance pH catheter (Medtronic Inc., Shoreview, MN). All studies were re-reviewed by a single investigator (MH). The catheter consisted of six impedance sensors positioned 3, 5, 7, 9, 15, and 17 cm from the lower esophageal sphincter (LES) and pH sensors 5 cm above (proximal channel) and 10 cm below the LES. Patients who underwent combined pH impedance monitoring were defined as having an abnormal study if they had any of the following findings on their esophageal pH impedance: esophageal acid exposure time of >4.5% and/or the detection of greater than 73 reflux episodes by impedance in a 24-hour period.

Reflux patients were further categorized into having upright, mixed, or supine predominant reflux. Patients were considered to have predominantly upright reflux if the percent time in upright acid exposure was >6.5% and <2.5% in supine acid exposure time. Patients who had >4.5% supine acid exposure time and <6.5% in upright acid exposure were considered to have predominantly supine reflux. If patients had both >6.5% upright and >2.5% supine time in acid exposure, they were considered to have mixed reflux. If patients were found to have reflux by impedance criteria, upright and supine reflux was defined as having >80% of impedance events in the upright or supine position, respectively. If these criteria were not met, then the patient was categorized as mixed. Patients with abnormal pH impedance studies were further characterized to having predominantly nonacid versus acid reflux based on previously described criteria [16].

### 2.4. Assessment of Severity of Idiopathic Subglottic Stenosis

The severity of the airway stenosis in patients with idiopathic subglottic stenosis was assessed by pulmonary function testing. Maximum voluntary ventilation (MVV) was used as a measure of airway patency. In patients who have had multiple pulmonary function tests done, both the lowest recorded value prior to airway intervention and the value taken closest to the time of the esophageal pH impedance study were obtained. This way, both the baseline stenosis value and the airway patency closest to time of reflux study could be assessed.

### 2.5. Statistical Analysis

Patients with abnormal and normal impedance studies were compared with two-tailed student *T*-testing and chi square as appropriate. Bivariate linear regression was performed to assess relationship between degree of reflux and airway stenosis severity. Continuous data was reported as mean ± standard deviation (SD), and categorical data was presented as a percentage of totals. Data was stored and analyzed with JMP 10.0 (SAS Institute Inc., Cary, NC). *P* < 0.05 was determined to be significant.

## 3. Results

### 3.1. Patient Demographics

We identified 262 patients with subglottic stenosis with or without tracheal stenosis who were seen in the clinic September 2010 through December 2016, with 159 given the diagnosis of ISS after thorough evaluation (ANCA-negative, no prior evidence of trauma, or other secondary causes identified). A total of 41 ISS patients were identified that fit within our inclusion criteria (Table 1). The mean age was 55 years, mean BMI was 31, and all but one patient were female (40/41; 98%). At the time of impedance study, 24 (59%) patient were on a PPI. 46% (19/41) of patients assessed were having symptoms of airway obstruction during their clinical evaluation. Only 11 of the 41 patients underwent EGD at our institution, and LA grade A esophagitis was noted in 1 patient, whereas the other 10 had no evidence of esophagitis.

### 3.2. Comparison of Patients with Reflux and without Reflux Detected by Esophageal pH Impedance

19 (46%) ISS patients were found to have abnormal pH impedance studies (Table 2). Among the 19 patients with abnormal pH impedance studies, the average esophageal acid exposure time was 8.9% compared to 0.9% among those with a negative study. 9 patients had abnormal pH impedance studies despite receiving PPI therapy during the study period, compared to 10 patients with abnormal impedance while off PPI therapy. Of the 9 patients with abnormal esophageal acid exposure, 4 had gastric acid present greater than 60% of the time despite taking 40 mg PPI daily (*n* = 2) or 40 mg PPI twice daily (*n* = 2). This indicates a suboptimal response to acid suppressive therapy due to hypermetabolism, suboptimal dosing, or noncompliance. The remaining 5 patients had adequate gastric acid suppression. There was no difference between patients with and without reflux in a number of acid exposures in the supine position (*P* = 0.1859). There was also no significant difference between BMI (*P* = 0.7544), presence of symptoms (*P* = 0.1715), PPI usage (*P* = 0.1763), and MVV (lowest: *P* = 0.5430; closest: *P* = 0.9435) between the two groups.

### 3.3. Characterization of Reflux Patterns in Patients with Abnormal pH Impedance Studies

**Figure 1:**
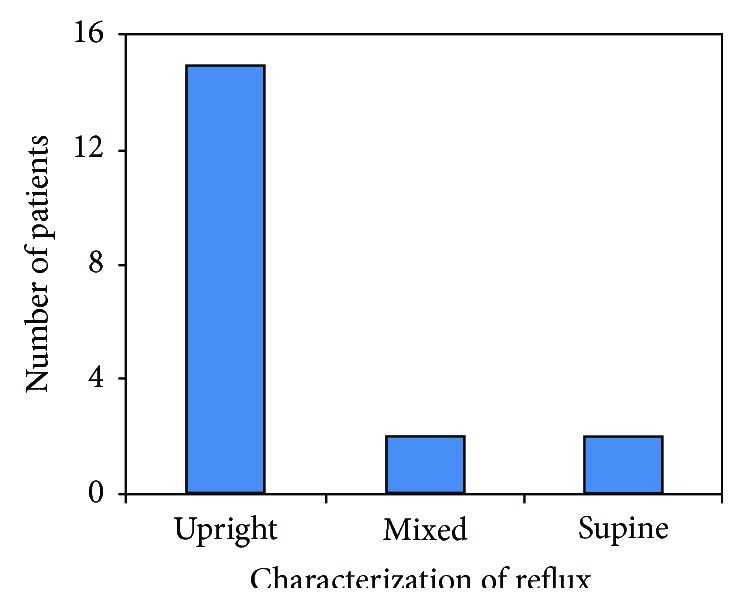
Distribution of GERD phenotypes.

**Figure 2:**
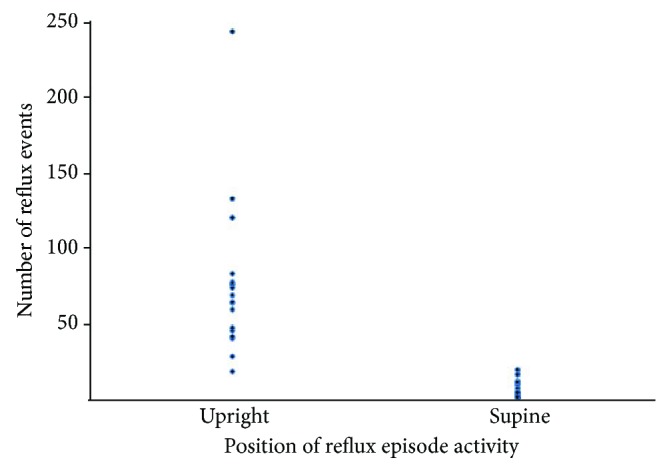
Number of reflux events among patients with upright and supine GERD phenotypes.

Of the 19 patients who were found to have an abnormal number of reflux events on impedance, 15 fit criteria for upright reflux, 2 for supine, and 2 for mixed (Figure 1). The distribution of reflux events as detected by impedance is shown in Figure 2. 15 patients had predominately acid reflux, while 4 had excessive nonacid reflux. Bivariate linear regression showed no association with the degree of reflux assessed by both total refluxes and total % time proximal channel acid exposure as compared to severity of ISS measured by the lowest MVV (total refluxes: *r*^2^ = 0.03; % time in proximal channel acid exposure: *r*^2^ = 0.004; data not shown) and closest MVV to reflux assessment (total refluxes: *r*^2^ = 0.002; % time in proximal channel acid exposure: *r*^2^ = 0.05; data not shown).

## 4. Discussion

While GERD has been implicated as an etiologic factor in idiopathic subglottic stenosis, the characterization of reflux frequency and patterns in this patient group is incompletely understood. Previous studies have implicated the role of gastroesophageal reflux in the development of ISS; however, several of these studies either had limited patient populations [6, 7] or did not exclude patients with secondary causes for subglottic stenosis [11]. Of the larger studies investigating patients with ISS, reflux was evaluated with pH testing only [5, 9], and as such were not able to identify patients with nonacid reflux.

To our knowledge, this is the first study which evaluated reflux patterns in a relatively large cohort of ISS patients using esophageal pH impedance analysis. This study produced two main findings. First, among patients with ISS at our institution, pathological GERD was found in close to 50% of patients referred for ambulatory pH impedance testing. Second, the reflux patterns among patients with ISS are almost exclusively upright GERD. In terms of GERD frequency, our findings are consistent with prior studies which have shown that a large proportion of patients with ISS have abnormal esophageal acid exposure. The novel finding of our study is that the GERD observed in this group occurs almost exclusively in the upright position. An upright pattern of GERD is generally thought to be less associated with esophageal injury such as Barrett's esophagus and esophagitis as the reflux occurs during daytime after meals, and not at night during sleep [17]. Supine reflux, however, occurs most often at night and is thought to lead to more esophageal injury due to delayed clearing in the absence of daytime swallowing and more proximal extent due to lack of gravitational protection which occurs in the upright position. Therefore, our finding that the GERD pattern in this patient group was predominantly upright is both surprising and raises important questions about the potential role of GERD in ISS. Future studies are needed to determine whether this reflux pattern may in fact be a result of an altered esophago-thoracic pressure gradient in ISS. It is noteworthy that close to 50% of patients with pathological GERD were prescribed a PPI at the time of the pH impedance study, although almost half of these had >60% gastric acid present during the study. Reasons for uncontrolled reflux despite PPI in our patients might therefore include lack of compliance, suboptimal dosing, or PPI hypermetabolism. Esophageal pH impedance monitoring while on therapy may therefore be valuable tool to ensure adequate acid suppression among patients with ISS who have documented GERD.

In our study, we found no correlation between the severity of ISS as measured by MVV and degree of reflux (Table 2), but this does not exclude the possibility that GERD may be the result of ISS in certain patients. In addition, 50% of patients had no pathological GERD detected, which indicates that GERD may not be a factor in many patients with ISS.

The strengths of this study include the relatively large sample size and the newer technology of pH impedance testing. However, this study also has several limitations. First, the retrospective nature of this study inherently introduces elements of bias, including the potential for selection bias. However, idiopathic subglottic stenosis is rare, and performing adequately powered prospective clinical or randomized studies is challenging. Our clinical practice encourages evaluation of GERD in most patients with ISS, but we cannot exclude referral bias as ISS patients with GERD symptoms or risk factors might be more likely to undergo esophageal testing. However, this would likely bias our results in the direction of overestimating the prevalence of GERD in this population. In addition, because several of the patients had prior surgical intervention due to severe airway constriction, the patient's true baseline severity of their ISS could not be fully assessed. Thus, our analysis to assess a correlation between the presence of GERD and ISS severity should be interpreted with caution. It has been noted in the past that morbidly obese patients with GERD had higher frequencies of upright reflux [18], which may point to obesity as the cause of this upright reflux pattern. However, in that study, comparisons were made between patients with a BMI between 35–39, 40–49, and >50. The patients in the BMI group of 35–39 have a higher frequency of upright reflux events, though none of them were noted to pathological reflux on pH monitoring. The mean BMI of our patients with reflux was around 31, and thus obesity alone likely does not account for our findings.

In conclusion, we have shown that in patients with idiopathic subglottic stenosis who have evidence of reflux detected by combined esophageal pH impedance testing, the majority of reflux in patients with ISS occurs in the upright position. These results challenge the current concept that GERD is the main pathogenic factor in patients with ISS and highlights the need for further prospective studies in this patient group.

## Figures and Tables

**Table 1:** Patient demographics.

	*N* = 41
Mean age (years)	54.8 ± 11.5
% female	97.6
BMI	30.9 ± 8.8
On PPI	24 (58.5%)
20 mg/day 30 mg/day 40 mg/day 60 mg/day 80 mg/day	1 1 11 1 10
Airway symptoms	46.3%

BMI: body mass index; PPI: proton pump inhibitor; airway symptoms: shortness of breath and dyspnea.

**Table 2:** Comparison between abnormal and normal pH impedance studies in idiopathic subglottic stenosis patients.

	Abnormal (*n* = 19)	Normal (*n* = 22)	*P* value
Acid exposure (pH)			
*Esophageal acid exposure (%)*			
Total	8.9 ± 6.4	0.9 ± 1.4	<0.0001
Upright	11.3 ± 9.5	1.25 ± 1.6	0.0002
Supine	4.8 ± 8.9	0.1 ± 0.3	0.0334
*Number of acid exposures*			
Total	84.9 ± 99.3	15.3 ± 14.0	0.0007
Upright	58.8 ± 33.3	13.2 ± 13.6	<0.0001
Supine	26.4 ± 77.1	2.0 ± 5.8	0.1859
*DeMeester score*	32.6 ± 26.0	3.6 ± 3.9	0.0001
Reflux episode activity (impedance)			
*Total*	81.47 ± 48.6	26.4 ± 18.1	0.0001
*Upright*			
Acid	49 ± 36.3	8.2 ± 9.0	0.0001
Nonacid	27.5 ± 59.3	15.0 ± 12.4	0.3775
*Supine*			
Acid	3.9 ± 5.0	0.7 ± 1.8	0.0148
Nonacid	1.1 ± 2.6	2.5 ± 5.7	0.3265
Airway symptoms	57.9%	42.1%	0.1715
BMI	30.4 ± 8.9	31.3 ± 8.9	0.7544
On PPI	47.4%	68.2%	0.1763
MVV			
*Lowest*	48.5 ± 19.7	44.6 ± 20.9	0.5430
*Closest to pH study*	56.5 ± 20.6	57.0 ± 21.9	0.9435

BMI: body mass index; PPI: proton pump inhibitor; MVV: maximum voluntary ventilation.

## Abbreviations

CI: Confidence interval
OR: Odds ratio
SD: Standard deviation
U: Units.
